# Activation of a Helper and Not Regulatory Human CD4+ T Cell Response by Oncolytic H-1 Parvovirus

**DOI:** 10.1371/journal.pone.0032197

**Published:** 2012-02-16

**Authors:** Olivier Moralès, Audrey Richard, Nathalie Martin, Dhafer Mrizak, Magalie Sénéchal, Céline Miroux, Véronique Pancré, Jean Rommelaere, Perrine Caillet-Fauquet, Yvan de Launoit, Nadira Delhem

**Affiliations:** 1 Institut de Biologie de Lille, UMR 8161, CNRS, Institut Pasteur de Lille, Université Lille-Nord de France, Lille, France; 2 Tumor Virology, Research Program Infection and Cancer, Deutsches Krebsforschungszentrum, Heidelberg, Germany; The University of Chicago, United States of America

## Abstract

**Background:**

H-1 parvovirus (H-1 PV), a rodent autonomous oncolytic parvovirus, has emerged as a novel class of promising anticancer agents, because of its ability to selectively find and destroy malignant cells. However, to probe H-1 PV multimodal antitumor potential one of the major prerequisites is to decipher H-1 PV direct interplay with human immune system, and so prevent any risk of impairment.

**Methodology/Principal findings:**

Non activated peripheral blood mononuclear cells (PBMCs) are not sensitive to H-1 PV cytotoxic effect. However, the virus impairs both activated PBMC proliferation ability and viability. This effect is related to H-1 PV infection as evidenced by Western blotting detection of H-1 PV main protein NS1. However, TCID50 experiments did not allow newly generated virions to be detected. Moreover, flow cytometry has shown that H-1 PV preferentially targets B lymphocytes. Despite seeming harmful at first sight, H-1 PV seems to affect very few NK cells and CD8+ T lymphocytes and, above all, clearly does not affect human neutrophils and one of the major CD4+ T lymphocyte subpopulation. Very interestingly, flow cytometry analysis and ELISA assays proved that it even activates human CD4+ T cells by increasing activation marker expression (CD69 and CD30) and both effective Th1 and Th2 cytokine secretion (IL-2, IFN-γ and IL-4). In addition, H-1 PV action does not come with any sign of immunosuppressive side effect. Finally, we have shown the efficiency of H-1 PV on xenotransplanted human nasopharyngeal carcinoma, in a SCID mouse model reconstituted with human PBMC.

**Conclusions/Significance:**

Our results show for the first time that a wild-type oncolytic virus impairs some immune cell subpopulations while directly activating a Helper CD4+ T cell response. Thus, our data open numerous gripping perspectives of investigation and strongly argue for the use of H-1 PV as an anticancer treatment.

## Introduction

Novel anticancer strategies aim at enhancing both tumor cell death and antitumor immune response through recognition of tumor antigens by the immune system [1], [2]. As new approaches for tumor targeting, replication-competent oncolytic viruses have been drawing special attention for a while because they exert specific antitumor effects in vivo [1], [3], [4], [5], [6].

More particularly, some autonomous parvoviruses, especially H-1 parvovirus (H-1 PV), are able to replicate in and kill both cancer cell lines and human primary cells cultured from patient tumor samples [7] while remaining harmless for normal cells. This remarkable feature, known as oncolysis, ultimately results in the decrease of spontaneous, chemically induced and xenografted tumor incidence in laboratory animals [2]. However, H-1 PV oncosuppression seems to involve more than just tumor cell killing. Indeed, H-1 PV may also exert a vaccination effect by enhancing effective immune response as several studies tend to suggest. For example, H-1 PV-mediated cell lysates stimulate antigen presentation by dendritic cells, leading to cytotoxic T lymphocyte activation and proinflammatory cytokine release [8]. Besides, H-1 PV *in vivo* antitumor effect has been reported to be related to both direct oncolysis and immune response triggering in a human hepatoma metastatic model [9]. More recently, immunocompetent rats carrying syngenic gliomas were shown to be more susceptible to H-1 PV oncosuppressive action than immunodeficient ones xenografted with human glioma cells. The former exhibited complete and stable cancer remission whereas the latter were only partially cured, confirming the existence of a major immunological component in H-1 PV therapeutic effect [10]. But none of these studies investigated the direct interplay between H-1 PV and immune system, particularly human immune cells.

With a view to using H-1 PV as an anticancer treatment, we propose to decipher H-1 PV effect on human immune cells to probe its multimodal antitumor potential as well as its harmlessness. As a first approach, we analyzed the effect of H-1 PV direct inoculation on peripheral blood mononuclear cells (PBMCs) under activation conditions or not. H-1 PV does not cause any major changes in non activated PBMCs. On the contrary, activated ones suffer both proliferation ability and viability impairment as well as significant lysis, while sustaining overactivation and both Th1 and Th2 type cytokine secretion. Very interestingly, further investigation indicated that H-1 PV does not only preserve CD4+ T cell subpopulation but also enhances its activation status as well as both IL-2 and IFN-γ secretion. Any effective immune response activation upon infection might get offset by the induction of a regulatory response which would result in effector T cell inhibition. So, CD4+CD25+ regulatory T cells were further analyzed because they are able to inhibit antitumor immune response and thus, are associated with the progression of cancer [11], [12]. These cells show neither viability nor proliferation or phenotypical alteration. Surprisingly, their suppressive activity is even inhibited upon H-1 PV infection. Taken altogether, our results confirm H-1 PV relevance in therapy by demonstrating *ex vivo* its direct immunomodulating potential, possibly resulting in improved antitumor immunity, without inducing Treg cell activation.

## Results

### PHA-activated PBMCs are affected by H-1 PV infection

For all the experiments performed on human PBMCs, cells were isolated from healthy donors' blood, PHA-activated or not, and inoculated with increasing amounts of purified H-1 PV (MOI 1, 5 and 25) or mock-treated from 24 h to 120 h. The impact of such treatments was first analyzed by assessing the distribution of PBMC main cell populations using flow cytometry (Table 1). After 24, 48 and 72 h of infection, cells were collected and labelled for appropriate cell surface antigens. As reported in Table 1, H-1 PV exerts no significant effect on the different cell populations when they are not activated, except for Natural Killer (NK) cell fraction whose frequency sustains a slight, viral dose-dependent decrease. However, under PHA stimulation conditions, B cells, CD8 T cells and NK cells rates seem to undergo a decrease that may be more or less significant depending on the cell type. Meanwhile, CD4+ T cells do not seem to be impaired by H-1 PV inoculation since their frequency remains remarkably stable under any experimental conditions.

**Table 1:** Main cell populations fractions within non activated or PHA-activated human PBMCs at 24, 48 and 72 h after H-1 PV infection.

Markers	Status	0 h p.i	24 h p.i				48 h p.i				72 h p.i			
			Mock	MOI 1	MOI 5	MOI 25	Mock	MOI 1	MOI 5	MOI 25	Mock	MOI 1	MOI 5	MOI 25
CD3CD4	NA	52,4	55,4	54	55	56,6	51,8	53,7	54,6	54,5	52,2	56	48,3	53,2
	**A**		**48,7**	**52,3**	**54,2**	**57,3**	**35,4**	**31,7**	**31,5**	**31,5**	**34,5**	**44,1**	**29,3**	**28,8**
CD3CD8	NA	10,7	17,1	16,4	18	17,9	22,9	16,2	24,4	17,2	19,9	19,9	46,3	13
	**A**		**29,4**	**31,7**	**30,4**	**28,3**	**26,5**	**24,5**	**23,3**	**27,4**	**18,2**	**14,4**	**3,85**	**5,72**
CD19CD20	NA	11,4	18,4	16,9	17,5	16,7	16,1	20,1	18,2	19,9	24,1	20	15,2	17,8
	**A**		**23,6**	**28,1**	**29,2**	**25,4**	**35,8**	**33,7**	**31,4**	**27,2**	**65,2**	**67,9**	**33,4**	**30**
CD3CD56	NA	5,25	4,82	4,57	4,73	3,39	4,34	2,69	2,97	2,65	4,27	2,71	2,36	1,34
	**A**		**4,81**	**5**	**4,67**	**3,39**	**4**	**4,49**	**4,36**	**3,41**	**3,5**	**4,08**	**2,61**	**1,24**
CD3CD30	NA	0,21	0	0	0,02	0,04	0,27	0	0,04	0,1	0,53	0,39	0,23	0,37
	**A**		**2,73**	**3,12**	**4,47**	**5,58**	**7**	**6,33**	**4,69**	**4,44**	**3,96**	**1,57**	**0,62**	**3,41**
CD3CD69	NA	0	0,23	0,18	1,28	0,58	0,04	0,3	0,56	0,51	0,33	0,68	0,43	0,55
	**A**		**1,96**	**1,7**	**1,65**	**0,95**	**24,7**	**20,8**	**18**	**17,1**	**12,4**	**16,3**	**28,5**	**34,3**

p.i: post-infection; NA: non activated PBMCs; A: activated PBMCs; MOI: multiplicity of infection.

Freshly isolated PBMCs were inoculated with increasing amounts of H-1 PV or mock-treated. For each condition, cells were labelled with appropriate antibodies and the fraction of each cell population was analyzed using flow cytometry. Results are expressed as percentages of total cells within PBMCs. CD3+CD4+, CD3+CD8+, CD19+CD20+ and CD3+CD56+ respectively indicate the % of CD4+ helper T cells, CD8+ effector T cells, B cells and NK cells within PBMCs. CD3+CD69+ and CD3+CD30+ respectively indicate the % of early and late activated T cells within PBMCs.

*Representative data for all markers.*

**Figure 1:**
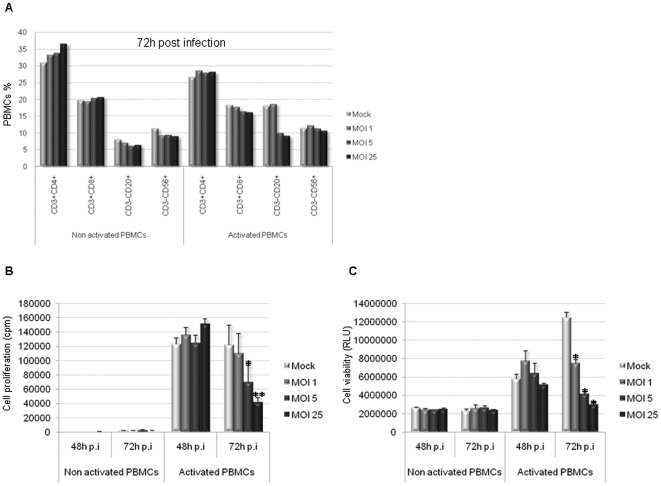
PHA-activated PBMCs are affected by H-1 PV infection. PBMCs were inoculated with increasing amounts of purified H-1 PV or mock-treated. MOI : multiplicity of infection, expressed as the number of plate-forming unit/cell; p.i : post-infection. **A**. *CD8+ effector T cell, B lymphocyte and NK cell populations are affected by H-1 PV infection within PHA-activated PBMCs, whereas CD4+ helper T cells are not*. Cells were collected 72 h p.i and labelled for appropriate cell surface antigens and the fraction of main lymphocyte populations within PBMCs was analyzed using flow cytometry. Results are expressed as percentages of total cells within PBMCs. **B**. *PHA-activated PBMC proliferation ability is decreased upon H-1 PV infection*. Cell proliferation was assessed by metabolic incorporation of tritiated thymidine into cellular DNA. Assays were performed at different times after H-1 PV inoculation but only 48 and 72 h p.i conditions are shown. Results from one representative experiment are represented as means of triplicate wells with ± standard deviation bars and expressed in count per minute (cpm). Statistical analysis for 4 independent experiments was performed using a Mann-Whitney test (* p<0,05; ** p<0,01 relative to Mock condition). **C**. *PBMCs viability is decreased upon H-1 PV infection*. Cell viability was evaluated using a test based on a bioluminescent reaction measuring the amount of ATP present in living cells. Assays were performed at different times after H-1 PV inoculation but only 48 and 72 h conditions are shown. Results from one representative experiment are represented as means of trilplicate wells with ± standard deviation bars and expressed in relative light unit (RLU). Statistical analysis for 4 independent experiments was performed using a Mann-Whitney test (* p<0,05 relative to Mock condition). *Representative data from 4 independent experiments*.

To validate the impact of H-1 PV infection on freshly isolated PBMCs, we have reproduced the 72 h p.i condition using PBMC from 4 different donors (Table 2 and Fig. 1A). Results obtained especially show that, within activated PBMCs, B lymphocyte rates are clearly decreased upon infection whereas NK cells and the CD8+ T lymphocytes are slightly decreased. However, concerning the CD4+ T lymphocytes, one can observe that the level is maintained throughout the infection.

**Table 2:** Main cell populations fractions within non activated or PHA-activated human PBMCs at 72 h after H-1 PV infection.

Markers	Status	0 h p.i	72 h p.i			
			Mock	MOI 1	MOI 5	MOI 25
CD3CD4	NA	24,67	31	33,25	33,8	36,63
	**A**		**26,7**	**28,58**	**28**	**28,2**
CD3CD8	NA	9,91	19,8	19,45	20,4	20,68
	**A**		**18,33**	**17,8**	**16,49**	**16,13**
CD19CD20	NA	4,35	8,145	7,22	6,25	6,54
	**A**		**18,10**	**18,70**	**10,03**	**9,20**
CD3CD56	NA	8,3	11,42	9,38	9,49	9,09
	**A**		**11,55**	**12,30**	**11,40**	**10,69**

p.i: post-infection; NA: non activated PBMCs; A: activated PBMCs; MOI: multiplicity of infection.

Freshly isolated PBMCs were inoculated with increasing amounts of H-1 PV or mock-treated. For each condition, cells were labelled with appropriate antibodies and the fraction of each cell population was analyzed using flow cytometry. Results are expressed as percentages of total cells within PBMCs. CD3+CD4+, CD3+CD8+, CD3-CD20+ and CD3-CD56+ respectively indicate the % of CD4+ helper T cells, CD8+ effector T cells, B cells and NK cells within PBMCs.

*Mean data of 4 independent experiments from 4 different donors.*

Infected PBMCs were further analyzed with regard to their proliferation ability and viability. On one hand, cell proliferation was assessed using a test based on the metabolic incorporation of tritiated thymidine into cellular DNA. We clearly observed that PHA-activated PBMC proliferation ability is dramatically impaired in a viral dose-dependent manner (Fig. 1B). On the other hand cell viability was measured using a test based on a bioluminescent reaction measuring the amount of ATP present in living cells. The results perfectly correlate with proliferation assays since they show a major virus-dependent decrease in PHA-activated PBMC viability upon H-1 PV infection, unlike non activated cells whose viability is preserved (Fig. 1C). Cell proliferation impairment is statistically significant 72 hours post-infection at MOI 5 and 25 (p<0,05) when compared to Mock condition and so is cell viability decrease whatever the MOI is (p<0,05).

### H-1 PV exerts its effect on PHA-activated PBMCs in an NS1-dependent manner

**Figure 2:**
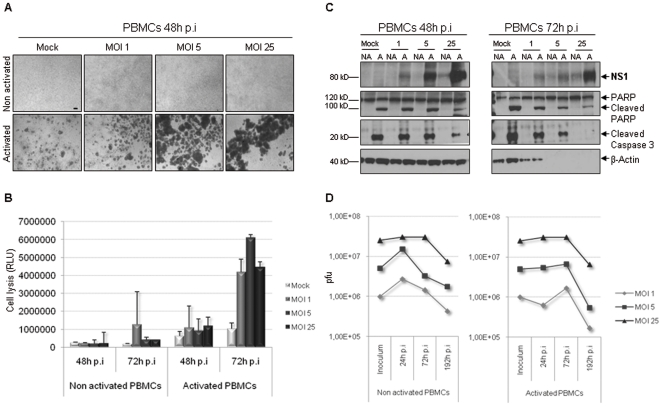
H-1 PV exerts its effect on PHA-activated PBMCs in an NS1-dependent manner. PBMCs were inoculated with increasing amounts of purified H-1 PV or mock-treated. MOI : multiplicity of infection, expressed as the number of plate-forming unit/cell; p.i : post-infection. **A**. *H-1 PV alters PHA-activated PBMC morphology*. For each condition, pictures of cells were taken in the course of infection but only those taken 48 h post-infection are shown. Scale bar = 100 µm. **B**. *PHA-activated PBMCs are lysed upon H-1 PV infection*. H-1 PV cytotoxicity was assessed using a test based on a bioluminescent reaction measuring the leak of a cellular marker from cytoplasm to culture medium, which reveals the loss of plasma membrane integrity. Assays were performed at different times after H-1 PV inoculation but only 48 and 72 h conditions are shown. Results are represented as means of triplicate wells with ± standard deviation bars and expressed in relative light unit (RLU). **C**. *PHA-activated PBMC death upon H-1 PV infection is related to NS1 protein expression but does not depend on caspase 3 activation*. Non activated (NA) and activated (A) PBMC total protein extracts were prepared in the course of infection. Equal amounts of proteins were separated by 4–12% SDS-PAGE and analyzed by Western blot for the presence of NS1 protein, cleaved caspase 3 and both full-length and cleaved PARP. β-actin was used as a loading control. **D**. *PBMCs do not support an H-1 PV productive infection*. Both PBMCs and culture supernatants were collected and tested for the presence of infectious viral particles using the Tissue Culture Infectious Dose 50 method. The graph represents the time course of both released and non-released virions amounts which were added and expressed in plaque forming unit (pfu). *Representative data from 3 independent experiments*.

For each condition, pictures of the cells were taken in the course of infection. An overall visual analysis showed that H-1 PV inoculation causes important morphological changes to PHA-activated PBMCs, leading to cell gathering in a viral dose-dependent manner (Fig. 2A). Cell aspect made us think of overactivation plates or cell death outward signs so we performed a cytotoxicity assay to settle on either possibility. The test, which is based on a bioluminescent reaction measuring the leak of a cellular marker from cytoplasm to culture medium, allows the loss of plasma membrane integrity to be revealed. 72 h after H-1 PV inoculation, there was a 4- to 6-fold increase in stimulated PBMC basal lysis whatever the dose of virus is (Fig. 2B). So H-1 PV seems responsible for killing PHA-activated cells, but possibly in an indirect way. At the same time, PBMC total protein extracts were prepared in the course of infection and analyzed by Western blotting. Non Structural protein 1 (NS1) plays a crucial role in H-1 PV life cycle achievement since it is involved in viral DNA replication, viral proteins expression and also cytotoxicity, allowing progeny virions to be released [13], [14], [15]. In this respect, we consequently searched for NS1 (76 kDa) in infected PBMCs. As shown in Figure 2C, PHA-activated PBMCs are able to produce NS1 in a viral dose-dependent manner. We also noticed that a slight amount of the protein becomes visible in non activated PBMCs under MOI 5 and 25 conditions. Both caspase 3 and PARP cleavage is characteristic of immune cells activation at a molecular level. It is also a well-known marker of ongoing apoptosis. In the context of H-1 PV-infected PBMCs, caspase 3 and PARP are indeed cleaved under activation conditions but their expression rate remains stable whatever infection conditions are (Fig. 2C). This suggests that PHA-activated PBMCs die in a caspase 3-independent manner upon H-1 PV infection. Besides, considering that H-1 PV typically induces the lysis of productively infected cells, we asked whether NS1 production was followed by the release of newly generated virions. PBMCs and culture supernatants were then collected and tested according to the TCID50 method in order to quantify intracellular and released virions. We were able to detect the presence of infectious viral particles. However, the quantity did not exceed the inoculum that was not removed from the cells, and even tended to decrease (Fig. 2D). This shows that PBMCs do not support an H-1 PV productive infection despite their ability to express NS1 protein.

### PHA-activated PBMCs are overactivated during H-1 PV infection and secrete Th1 and Th2 type cytokines

**Figure 3:**
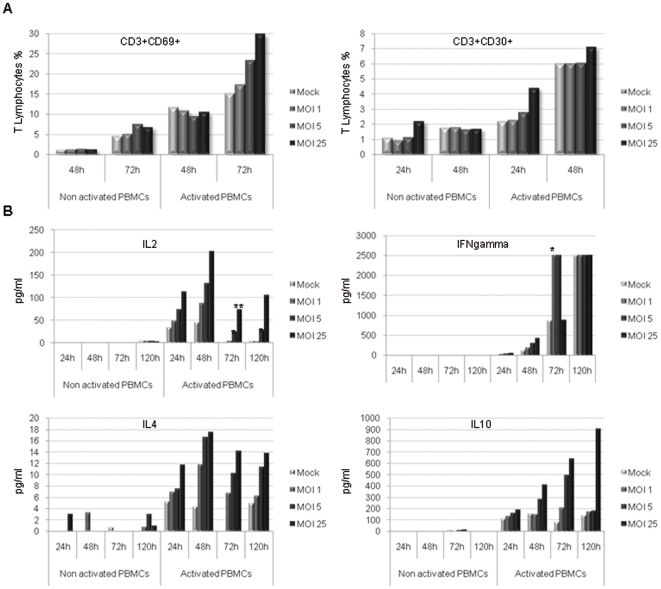
PHA-activated PBMCs are overactivated during H-1 PV infection and secrete Th1 and Th2 type cytokines. PBMCs were inoculated with increasing amounts of purified H-1 PV or mock-treated. MOI : multiplicity of infection, expressed as the number of plate-forming unit/cell. **A**. *Early and late activation marker expression is increased at the surface of PHA-activated PBMCs upon H-1 PV infection*. In the course of infection, cells were collected, labelled for the expression of early (CD69) and late (CD30) activation markers and analyzed using flow cytometry. Results are expressed as percentages of activated cells within T lymphocytes (CD3+ cells). **B**. *H-1 PV enhances the ability of PHA-activated PBMCs to secrete Th1 and Th2 type cytokines*. In the course of infection, culture supernatants were collected and tested for cytokine detection by enzyme-linked immunosorbent assay (ELISA). Results are expressed in pg/ml as means of duplicate wells and after subtraction of the background value. Statistical analysis for 3 independent experiments was performed using a Mann-Whitney test (* p<0,05; ** p<0,01 relative to Mock condition). *Representative data from 3 independent experiments*.

Cells were collected and CD3+ T cells were analyzed for the expression of early (CD69) and late (CD30) activation markers using flow cytometry. In a very interesting manner, T lymphocytes (CD3+) keep their ability to get activated upon H-1 PV infection. The virus even potentiates it, as evidenced by an increase in the expression of respectively early and late activation markers CD69 and CD30. Results show that CD69 and CD30 expression is increased at the surface of activated CD3+ T cells in a viral dose-dependent way (Fig. 3A). Moreover, culture supernatants were collected and tested for cytokine detection using Enzyme-Linked ImmunoSorbent Assay (ELISA) in order to assess the time course of PBMC cytokine release. We observed that H-1 PV enhances PHA-activated PBMC ability to secrete Th1 cytokines such as IL-2 and IFN-γ (Fig. 3B) but also Th2 cytokines, namely IL-4 and IL-10 (Fig. 3B). Interestingly, H-1 PV is also able to induce non activated PBMC late IL-4 secretion (120 h after the inoculation). Remarkably, these increased cytokine secretions depend on the viral dose and some of them remain detectable up to 120 h after H-1 PV inoculation.

### Unlike PBMCs, CD4+ T cells do not undergo any major impairment upon H-1 PV infection

**Figure 4:**
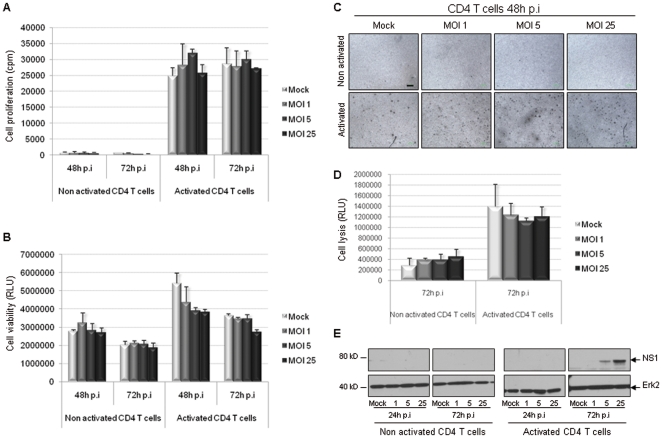
Unlike PBMCs, CD4+ T cells do not undergo any major impairment upon H-1 PV infection. CD4+ T cells were inoculated with increasing amounts of H-1 PV or mock-treated. MOI : multiplicity of infection, expressed as the number of plate-forming unit/cell; p.i : post-infection. **A**. *CD4+ T cell proliferation ability is not altered upon H-1 PV infection*. Cell proliferation was assessed by metabolic incorporation of tritiated thymidine into cellular DNA. Only 48 and 72 h p.i conditions are shown. Results are represented as means of triplicate wells with ± standard deviation bars and expressed in count per minute (cpm). **B**. *H-1 PV does not affect CD4+ T cell viability*. Cell viability was evaluated using a test based on a bioluminescent reaction measuring the amount of ATP present in living cells. Only 48 and 72 h p.i conditions are shown. Results are represented as means of triplicate wells with ± standard deviation bars and expressed in relative light unit (RLU). **C**. *Infection of CD4 T cells by H-1 PV is not related to any morphological changes*. For each condition, pictures of cells were taken in the course of infection but only those taken 48 h after H-1 PV inoculation are shown. Scale bar = 100 µm. **D**. *H-1 PV does not modify CD4+ T cell basal lysis*. H-1 PV cytotoxicity was assessed using a test based on a bioluminescent reaction measuring the leak of a cellular marker from cytoplasm to culture medium, which reveals the loss of plasma membrane integrity. Results are represented as means of triplicate wells with ± standard deviation bars and expressed in relative light unit (RLU). **E**. *CD4+ T cells transiently express a slight amount of NS1 protei*n. Equal amounts of total CD4+ T cell proteins were separated by 4–12% SDS-PAGE and analyzed by Western blot for the presence of NS1 protein. Erk2 was used as a loading control. *Representative data from 2 independent experiments*.

For all the experiments performed on human CD4+ T cells, PBMCs were first isolated from healthy donors' blood. Then CD4+ T cells were negatively selected, activated (or not) using both anti-CD3 and anti-CD28 antibodies and inoculated with increasing amounts of H-1 PV (MOI 1, 5 and 25) or mock-treated from 24 h to 120 h. First, we analyzed infected CD4+ T cells in terms of proliferation ability and viability as described above for PBMCs. The results showed that CD4+ T cell proliferation ability is not altered upon H-1 PV infection whether cells are activated or not (Fig. 4A). In the same manner, H-1 PV does not affect CD4+ T cell viability (Fig. 4B). Slight differences between the subgroups concerning both cell proliferation and viability were not statistically significant (p>0,1). Moreover, pictures of the cells were taken in the course of infection and showed that H-1 PV inoculation is not related to any major morphological changes (Fig. 4C). Besides, cytotoxicity assays did not prove any increase in non activated and activated CD4+ T cell basal lysis (Fig. 4D). Nevertheless, at a molecular level, Western blot analysis revealed that CD4+ T cells transiently express a slight, dose-dependent amount of NS1 protein.

### H-1 PV preserves or even enhances non-activated and activated CD4+ T cell secretion ability

**Figure 5:**
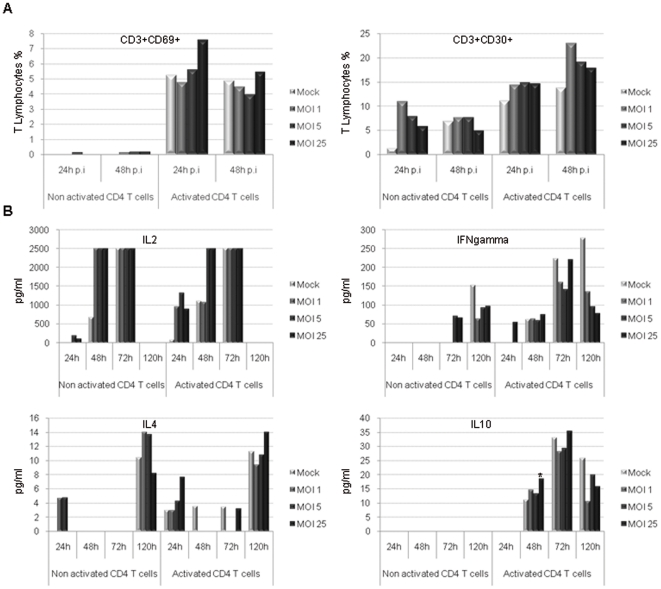
H-1 PV preserves or even enhances non-activated and activated CD4+ T cell secretion ability. CD4+ T cells were inoculated with increasing amounts of purified H-1 PV or mock-treated as described in Materials and Methods section. MOI : multiplicity of infection, expressed as the number of plate-forming unit/cell; p.i : post-infection. **A**. *Early and late activation marker expression is increased at the surface of CD4+ T cells upon H-1 PV infection*. In the course of infection, cells were collected, labelled for the expression of early (CD69) and late (CD30) activation markers and analyzed using flow cytometry. Results are expressed as percentages of activated cells within CD4+ T cell population. **B**. *H-1 PV enhances CD4+ T cell ability to secrete Th1 and Th2 type cytokines*. In the course of infection, culture supernatants were collected and tested for cytokine detection by enzyme-linked immunosorbent assay (ELISA). Results are expressed in pg/ml as the mean of duplicate wells and after subtraction of the background value. Statistical analysis for 2 independent experiments was performed using a Mann-Whitney test (* p<0,05 relative to Mock condition). *Representative data from 2 independent experiments*.

In the course of infection, CD4+ T cells were collected and analyzed for the expression of early CD69 and late CD30 activation markers using flow cytometry. Activated CD4+ T cell expression of both markers is increased from the beginning of infection (Fig. 5A). And interestingly, CD30 expression is also enhanced in infected, non activated CD4+ T cells (Fig. 5A). At the same time, culture supernatants were tested for cytokine detection using ELISA method. CD4+ T cells keep their ability to secrete Th1 and Th2 cytokines (IL-2, IFN-γ, IL-4 and IL-10) (Fig. 5B). Very interestingly, H-1 PV is even able to enhance activated CD4+ T cell ability to release IL-2 right from the beginning of the infection, and IL-4 at early and late infection stages (24 h and 120 h after the inoculation) (Fig. 5B). Noticeably, H-1 PV also induces IL-2 secretion in non activated CD4+ T cells from 24 to 72 h after the inoculation (Fig. 5B).

### Unlike PBMCs, neutrophils cells do not undergo any major impairment upon H-1 PV infection

**Figure 6:**
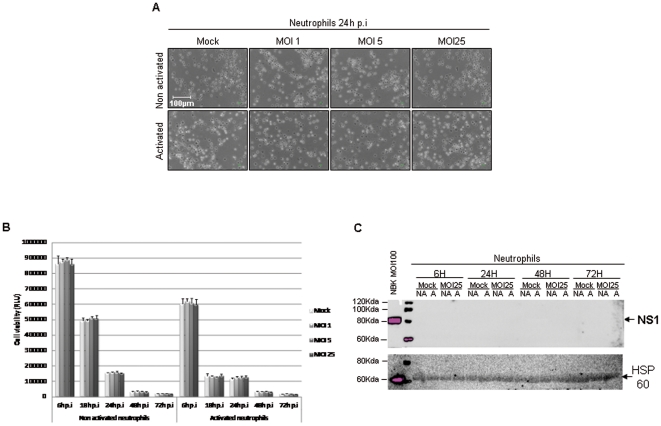
H-1 PV does not affect neutrophils viability. Human neutrophils cells were inoculated with increasing amounts of purified H-1 PV or mock-treated. MOI : multiplicity of infection, expressed as the number of plate-forming unit/cell; p.i : post-infection. **C**. *Infection of Neutrophils by H-1 PV is not related to any morphological changes*. For each condition, pictures of cells were taken in the course of infection but only those taken 48 h after H-1 PV inoculation are shown. Scale bar = 100 µm. **B**. *H-1 PV does not affect Neutrophils viability*. Cell viability was evaluated using a test based on a bioluminescent reaction measuring the amount of ATP present in living cells. 6 h, 18 h, 24 h, 48 h and 72 h p.i conditions are shown. Results are represented as means of triplicate wells with ± standard deviation bars and expressed in relative light unit (RLU). *Representative data from 5 independent experiments*.

Isolated neutrophils cells were LPS-activated or not (1 µg/ml), and inoculated with increasing amounts of purified H-1 PV (MOI 1, 5 and 25) or mock-treated from 24 h to 72 h. The impact of such treatments was first analyzed by pictures of the cells which were taken in the course of infection. An overall visual analysis showed that H-1 PV inoculation is not related to any major morphological changes (Fig. 6A).

Then, we analyzed infected neutrophils in terms of viability as described above for PBMCs and CD4+ T cells. Although we observed a decrease in the viability of neutrophils 18 h after infection, it is not related to an effect of H-1PV but to the fact (and it is well documented) that isolated neutrophils can not be kept very longer in culture. Thus, H-1 PV does not affect neutrophil viability whatever the MOI and up to 72 hours of contact (Fig. 6B).

Finally, we confirmed that infected neutrophils did not express NS1 protein even transiently, confirming the complete absence of H-1 PV effect on this immune sub-population (Fig. 6C).

### H-1 PV does not affect Treg cell phenotype and viability while inhibiting their suppressive activity

**Figure 7:**
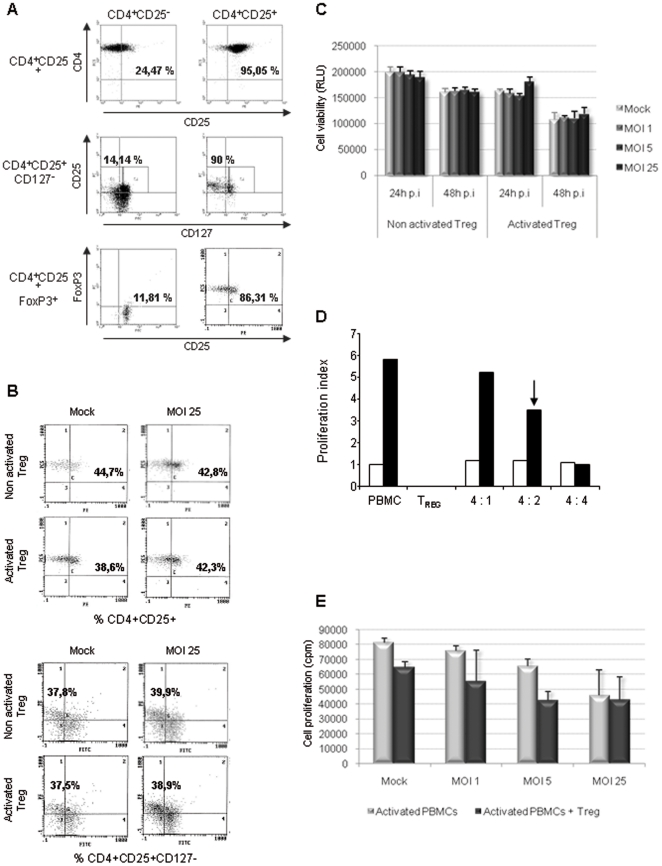
H-1 PV affects neither Treg cell phenotype nor viability while inhibiting their suppressive activity. Treg cells were inoculated with increasing amounts of purified H-1 PV or mock-treated. MOI : multiplicity of infection, expressed as the number of plate-forming unit/cell; p.i : post-infection. **A**. *Control of Treg cell purity*. Cells were labelled for Treg cell markers and analyzed using flow cytometry. Results are expressed as percentages of CD4+CD25+, CD4+CD25+CD127- and CD4+CD25+FoxP3+ cells within the whole cell population. **B**. *Treg cells do not undergo any major phenotypical changes upon infection*. Cells were labelled for Treg cell markers and analyzed using flow cytometry 48 h p.i. Results are expressed as percentages of CD4+CD25+ and CD4+CD25+CD127- cells within the whole cell population. **C**. *H-1 PV does not affect Treg cell viability*. Cell viability was assessed 24 and 48 h p.i using a bioluminescent test measuring ATP amount in living cells. Results are represented as means of triplicate wells with ± standard deviation bars and expressed in relative light unit (RLU). **D**. *Ex vivo Treg cells are able to suppress autologous activated PBMCs proliferation*. Autologous PBMCs were activated (anti-CD3 and -CD28 antibodies) and cultured with increasing quantities of Treg cells. PBMCs proliferation was assessed by metabolic incorporation of tritiated thymidine into cellular DNA 48 h p.i. Results are represented as means of triplicate wells with ± standard deviation bars and expressed in count per minute (cpm). The black arrow indicates the ratio chosen for further experiments. **E**. *H-1 PV is able to inhibit Treg cell suppressive activity*. Autologous PBMCs were activated (anti-CD3 and -CD28 antibodies), cultured with Treg cells (4 PBMCs for 2 Treg cells) and mock- or H-1PV-treated. PBMC proliferation was assessed by metabolic incorporation of tritiated thymidine into cellular DNA 48 h p.i. Results are represented as means of triplicate wells with ± standard deviation bars and expressed in count per minute (cpm). *Representative data from 3 independent experiments*.

CD4+CD25+ regulatory T cells (Treg) play an indispensable role in maintaining immunological unresponsiveness to self antigens and in suppressing excessive immune responses deleterious to the host. In the particular context of cancer, Treg cells have been increasingly demonstrated to play an active and significant role in the pathology progression and suppress antitumor-specific immunity. Thus, assessing Treg cell status has become a crucial point in the area of new clinical strategies development [11], [12]. So, we investigated the impact of H-1 PV on both regulatory T cell phenotype and function. Treg cells were isolated from healthy donors PBMCs and extemporaneously analyzed for their purity rate using flow cytometry. For this purpose, we assessed the expression of Treg cell-associated proteins, namely CD25 (IL-2 receptor α-chain), CD127 (IL-7 receptor α-chain) and FoxP3 (a transcription factor) [16]. CD4 and CD25 high expression defines cell purity which is superior to 95%. CD4+CD25− cell fraction slightly expresses CD25 or even does not at all (Fig. 6A). Lack of CD127 expression has recently been described to be characteristic of functional Treg cells. In contrast, CD4+CD25− cells do express this molecule. As shown in Figure 7A, we confirmed that 90% of CD4+CD25+ cells but only 14% of CD4+CD25− cells do not express CD127. In the same way, the analysis of intracellular FoxP3 expression shows that 86% of freshly isolated Treg cells express this protein whereas only 12% of CD4+CD25− cell fraction do. As previously described for PBMCs and CD4+ T cells, Treg cells were then infected with increasing amounts of H-1 PV or mock-treated for 24 or 48 h. The cells were first analyzed by flow cytometry to assess the impact of such treatments on Treg cell phenotype. The results are reported in Figure 7B and reveal no significant differences in the expression of Treg cell-associated markers, whether the cells are activated or not. Once again cell viability was evaluated using the same bioluminescent test which shows that H-1 PV inoculation does not affect activated or non activated Treg cell survival (Fig. 7C).

Regulatory T cells are known to be anergic upon anti-CD3 and anti-CD28 antibodies *in vitro* stimulation whereas PBMCs are able to proliferate under these conditions. The functional suppressive ability of freshly isolated Treg cells was assessed using a Mixed Leukocyte Reaction (MLR) test between autologous PBMCs and CD4+CD25+ cells at different ratios, and PBMC proliferation was determined after 48 h. Cell proliferation was measured using metabolic incorporation of tritiated thymidine into cellular DNA. Ex vivo human Treg cells still exert their suppressive activity in a dose-dependent manner, inhibiting up to 80% of PBMC proliferation (Fig. 7D). Regarding these results, we chose to perform our experiments under the 4 PBMCs to 2 Treg cells ratio (arrow, Fig. 7D) and investigated whether H-1 PV could affect Treg function. The MLR test was slightly adjusted to respond to our hypothesis since we performed it using autologous PBMCs and Treg cells, with or without H-1 PV. Under basal conditions (i.e. no stimulation and no virus) Treg cells are able to inhibit PBMC proliferation up to 30%. When H-1 PV is added, an additional impairment is expected according to the data depicted above. We indeed noticed an additional decrease of PBMC proliferation, especially under MOI 5 condition but very interestingly this decrease is weaker than expected when cells are infected with a higher amount of virus (MOI 25) (Fig. 7E). This strongly suggests that H-1 PV may inhibit Treg cell-mediated suppressive activity.

Other possible alterations of Treg cell activity were also evaluated. More particularly ELISA assays indicate that Treg cell cytokine pattern (IL-2, IFN-γ, TGFβ and IL-10) remains unchanged after 48 h of infection (data not shown). Besides, H-1 PV does not modify the anergic condition of Treg cells upon anti-CD3 and anti-CD28 activation. Massive IL-2 amounts combined with co-culture with irradiated autologous PBMCs are the only treatment which allows Treg cells to proliferate. Under these culture conditions we did not show any significant difference in Treg cell proliferation ability either (data not shown).

### H-1 PV is able to slow down and stabilize the growth of nasopharyngeal carcinoma in humanized SCID mice

**Figure 8:**
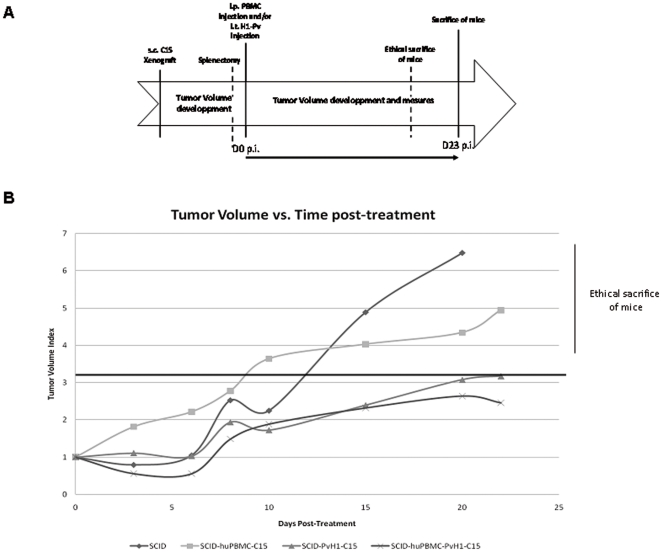
H-1 PV is able to slow down and stabilize the growth of nasopharyngeal carcinoma in humanized SCID mice. Immunodeficient SCID mice (Severe combined Immunodeficiency) were subcutaneously xenotransplanted with tumors of nasopharyngeal carcinoma (NPC) induced by C15 cells (human primary tumor cells of NPC). **A**. Timeline which summarizes the experimental protocol followed. After development of visible tumors, SCID mice were splenectomized and reconstituted (i.p) or not with 50.10^6^ human PBMC. At the same moment they also received or not H-1PV directly injected within the tumor. SCID mice received only one injection of H-1 PV throughout the protocol. **B**. Results are presented in Tumor Volume Index versus days post-treatment. There is a progressive increase in tumor size for the four groups of mice. Only the groups, which did not receive H-1 PV, saw their tumors grow to 6–7 times the original size. ***H-1 PV stabilize the growth of nasopharyngeal carcinoma in humanized SCID mice***. In two groups of mice that received H-1 PV, the growth of the tumor does not exceed a maximum of three times the size of the primary tumor. The size of these tumors stabilized to stay below the maximum threshold of 3. *Representative data from 2 independent experiments*.

The results are presented in Tumor Volume Index versus days post-treatment in Figure 8B. Although we have seen a progressive increase in tumor size for the four groups of mice, only the groups, which did not receive H-1 PV, saw their tumors grow to 6–7 times the original size. In addition, it seems that reconstitution with a simple immune system does not limit this growth (Fig. 8B).

In contrast, in two groups of mice that received H-1 PV, we see indeed increased tumor size up to 20 days after treatment. However, we showed that this growth does not exceed a maximum of three times the size of the primary tumor, as previously described in the work of Jean Rommelaere's team [34]. In addition and very interestingly, we observe that the size of these tumors stabilized to stay below the maximum threshold of 3.

Furthermore, we found a small decrease in tumor size in the group of mice reconstituted by human PBMC. This decrease in the presence of PBMC was reproducible. Unfortunately, the large variability in the initial tumor size did not allow us to validate statistically this decrease. In addition, we observed no tumor growth in all mice that had developed a very small tumor (difficult to measure) and who received H-1 PV regardless of immune reconstitution.

## Discussion

Oncolytic viruses, such as H-1 parvovirus, are currently considered serious candidates for cancer therapy because they exhibit antitumor properties *in vivo* [2], [4]. The immunomodulating potential of such anticancer agents could allow effective and less aggressive treatments to be developed. However, whether H-1 PV effects are also associated with the induction of specific immune responses that could potentiate its oncosuppressive properties remains to be defined. The present study shows for the first time that H-1 PV is able to directly induce an effective immune response, which could enhance antitumor immunity, in addition to its direct inherent oncolytic effect.

H-1 PV exerts several significant effects on PBMCs, but mostly when they are activated. Whether activated PBMCs are sensitive to H-1 PV infection is a hard question to be answered, depending on the criteria to meet in order to satisfy this little-defined notion. Basically, our results emphasize three major points : i) H-1 PV induces some viral dose-dependent events : NS1 protein expression and a decrease in both activated PBMC viability and proliferation ability, ii) the infection is associated with cytolysis in a dose-independent manner, and iii) newly generated infectious particles were not detected. H-1 PV life cycle initiation relies on host cell ability to activate early P4 promoter which is known to drive NS1 protein expression [17]. This ability has been described to be mostly exerted by transformed cells as they exhibit a specific expression pattern which is appropriate to achieve H-1 PV whole life cycle [18]. Nonetheless, NS1 protein detection proves that activated PBMCs allow viral life cycle to be initiated. Even though proliferating PBMCs are normal cells, our result is consistent with the fact that early steps of H-1 PV life cycle, notably P4 activation, are strongly dependent on host cell ability to enter S phase [19]. Moreover, strong ectopic NS1 protein expression has recently been reported to cause a significant decrease in normal human fibroblast viability [20], showing that transformation is not a compulsory requirement for NS1 to affect cells. This statement leads us to suggest that NS1 protein is responsible for PBMC viability impairment. The ability of infected, activated PBMC to proliferate also suffers a major decrease. This drop could result from NS1 protein ability to block cell cycle at G2 stage [14], [21]. But this hypothesis cannot be validated for sure as we did not assess cell cycle progression. NS1 properties also result in H-1 PV cytotoxicity [14], [22]. Even though PBMC lysis is clearly linked to the virus, the dose-independence of this phenomenon makes NS1 protein unlikely to be responsible for it. We suggest that both viability impairment and cytotoxicity should be uncoupled. In activated PBMCs, NS1 protein exerts probably more cytostatic than cytolytic properties while cell lysis would rather be an indirect effect occurring under any H-1 PV infection conditions and mediated by immune cells. In the context of cancer treatment, cytolysis should not be considered insurmountable for H-1 PV to be used since this effect is only partial. Moreover, H-1 PV-induced impairments of human immune cells are observed in an *ex vivo* context. But in a living organism, a pool of haematopoietic stem cells continually renew them and would then also recover the cell loss due to H-1 PV infection. Besides, we did not detect any newly generated virions, which would allow the administration of the agent to be controlled in therapy. Further investigation would be necessary to determine which steps of H-1 PV life cycle activated PBMCs are not able to sustain. Because activated PBMCs display an appropriate environment for H-1 PV to exert some of its properties, they can be considered sensitive to the virus but only partly and in an abortive way since they do not achieve the whole viral life cycle. Despite these impairments, activated, H-1 PV-infected PBMCs get overactivated, as pointed out by the overexpression of CD69 and CD30. They also mostly keep, or even enhance, their cytokine secretion, mainly the Th1 pathway. This strongly suggests that H-1 PV is able to induce an effective immunological response without any other in-between stimulation.

In contrast to PBMCs, CD4+ T cells do not undergo any significant changes upon infection and thereby, are clearly resistant to H-1 PV despite slight NS1 synthesis. This suggests that a threshold has to be reached for NS1 to exhibit its properties and/or that NS1 required post translational modifications are not achieved [13], [15], [22], [23]. Besides both unaltered viability and proliferation, CD4+ T cells remain or become activated and cytokine secretion-competent upon H-1 PV infection. Currently, CD4+ T cells are described as playing a key role in immune responses [24]. More particularly, CD4+ T helper responses (Th1 and Th2) are increasingly proving to mostly support antitumor immunity [25], [26], which is what we reported upon H-1 PV infection. Indeed, IL-2 secretion increases and IFN-γ remains detectable, thus demonstrating that Th1 pathway is induced. This is of special significance because Th1 response is strongly involved in antitumor immunity [27] and usually associated with the activation of (i) the major effector cell subpopulation, namely CD8+ T cells and (ii) the commitment of CD4+T cell into cytotoxic T cells[24], [28]. Even though CD8+ T cells seem to be targeted by H-1 PV within PBMCs, they are not totally cleared upon infection. We suggest that CD8+ T cell cytotoxic properties may be responsible for the cytolysis we reported on infected PBMCs. On the other hand, naive CD4+ T cells were very recently shown to be able to differentiate into Th1 cytotoxic T cells that display the remarkable ability to eradicate on their own an established human melanoma [29]. Thus, early IL-2 induction reported here might have induced IFN-γ release, and then CD4+ T cells to commit into effector T cells. This highlights another crucial aspect of CD4+ T cells in tumor clearance and their preserved functionality upon infection strengthens H-1 PV value as a promising candidate in anticancer therapy [27]. Besides its role in Th1 pathway and therefore antitumor immunity, IL-2 could also be used as a quantifiable biomarker of T cell activation. Such a sustained secretion of IL-2 could allow H-1 PV vaccination effect to be easily monitored using ELISA or ELISPOT assays [30]. Even though Th1 pathway is clearly the one to be focused on to target antitumor immunity, Th2 response is far from being insignificant and has been pointed out in anticancer therapy since specific antibodies can efficiently clear cancer cells [26], [31], [32]. Very interestingly, this pathway also seems to be initiated upon H-1 PV infection, as suggested by IL-4 release.

And very recently, in parallel with our *in vitro* study on human immune cells, Grekova S *et al* [33] aimed to establish whether immunomodulating mechanisms participate in the recently reported therapeutic potential of parvoviruses against pancreatic carcinoma in a rat model. In this work, they extended their study to pancreatic cancer and could show that this immunity can be further transferred to naïve tumor-bearing recipients. Recently they found that treatment of rat pancreatic tumors with H-1 PV resulted in the appearance of viral transcript in the PDAC (Pancreatic ductal adenocarcinoma)-draining celiac lymp nodes, with expression lasting up to 10 days post virus inoculation [34]. This involvement of lymphoid tissues in parvoviral oncosuppression is further sustained by their last study which showed that intratumoral treatment of PADC with H-1 PV led to the enrichment of these tissues with T-cells, with bias toward a Th1 response as evidence by the marked induction of IFN-γ expression. Moreover, they observed a splenomegaly which seems to be in part related to a general lymphocyte proliferation reaction, in response to viral infection. All these results recently obtained, in a rat model of pancreatic cancer [33] are in agreement with our results. Dissecting in more details the relation between H-1PV and the human immune system led to the consistent finding that H-1 PV can cause an increase in the CD4/CD8 ration both in rats and humans and induce Th1 expression in both species. In addition, all the experiences we have made on neutrophils show that H1-PV has no deleterious effect on viability, proving that we can definitively exclude a negative effect of PVH-1 on neutrophils.

Immune cell infection by H-1 PV leads to the induction of events which are characteristic of a broad, complementary and effective antitumor response. However, such a response is likely to be countered by the concomitant involvement of immunoregulatory components. To investigate this aspect, we focused our attention on CD4+CD25+ regulatory T cells, which turn out to be the main mediators of active immune escape in cancer. In this context, Treg cells have been shown to play a major role in pathology progression as they are able to suppress antitumor immune response [11]. Thus, assessing Treg cells status has become a crucial point in the area of new clinical strategies development. Treg cell phenotype was not altered upon H-1 PV infection and neither was their proliferation nor viability, whether they are activated or not. Treg cells are known to be major producers of IL-10 which is notably the lead immunosuppressive cytokine [35]. Besides, experiments performed on activated PBMCs showed a significant dose-dependent increase in IL-10 release upon infection (Fig. 3). Yet, it cannot be attributed to Treg cells as H-1 PV does not modify their cytokine secretion pattern (data not shown). However, IL-10 is also involved in humoral immune response enhancement together with other cytokines *in vivo* and monocytes/macrophages are likely to secrete high amounts of IL-10 when they are stimulated [36]. Even though we did not study H-1 PV direct interplay with these cells, this hypothesis is consistent with the fact that Th1 type cytokines are induced upon infection, possibly leading to monocyte/macrophage activation. Moreover, Treg cell inoculation with high amounts of virus seems to impair Treg cell ability to suppress PBMC proliferation. Then, it is tempting to assume that in an *in vivo* context, H-1 PV would not only be able to induce an effective immune response but also prevent the immunosuppressive tumor microenvironment from getting worse.

Concerning the results obtained in our *in vivo* model, they allow us to conclude that H-1 PV is able to slow down and stabilize the growth of nasopharyngeal carcinoma when injected in large tumors (13 mm^3^ to 210 mm^3^). While it completely prevents tumor growth when injected in the early stages of tumor development (tumor not measurable).

Regarding the addition of an immune system in this model, it appears that the PBMC are able to improve slightly decreased tumor growth. However, this small but reproducible non-significant decrease is not sufficient to conclude that the activation of CD4+ T induced by H-1 PV promotes the onco-suppressor effect of this oncolytic virus. In contrast, activation of CD4+ T can have an important complementary role in the anti-tumor response. Indeed, it is well known that activated CD4+ T can promote dialogue between immune cells of the innate or adaptive immunity, including the expansion of memory clones, lytic mechanisms or induction of apoptotic mechanisms.

In conclusion, our results show for the first time that H-1 parvovirus displays a cytostatic and cytolytic effect on some immune cells subsets, mostly upon activation conditions, while preserving and directly activating crucial CD4+ T cells after a single inoculation. Our approach is also particularly original since no study in the field of parvovirotherapy has been focused on the key aspect of immunosuppression for which we did not evidence any sign of activation upon H-1 PV infection. Therefore, our study proposes an optimal way of testing and proving H-1 PV remarkable immunomodulating properties. With this regard, H-1 oncolytic parvovirus perfectly meets the criteria for the use of a virus as an anticancer treatment.

## Materials and Methods

### Cells

Blood cells were isolated from 17 healthy donors. Each donor is associated to one particular cell population, except for 3 of them whose PBMCs as well as CD4+ T cells were isolated.

#### PBMC isolation

Peripheral Blood Mononuclear Cells (PBMCs) from healthy donors were isolated by standard density gradient centrifugation using Ficoll-Paque PLUS (Amersham Biosciences, Uppsala, Sweden). Donor blood was provided by the Etablissement Français du Sang – Nord de France (EFS), in accordance with the official ethics agreement between the latter and the Centre National de la Recherche Scientifique (CNRS) – Délégation Nord Pas-de-Calais et Picardie. The study was approved by the Institut de Biologie de Lille (CNRS) and EFS Institutional Review Boards, and informed consent was obtained in writing for each donor. When appropriate, CD4+ T cell and CD4+CD25+ cell isolation was further performed.

#### CD4+ T cell isolation

CD4+ T cells were isolated from PBMCs using a negative selection protocol according to manufacturer's instructions (Miltenyi Biotec, Berlin, Germany). Briefly, PBMCs are incubated for 10 minutes with a cocktail of biotinylated antibodies directed against CD8, CD14, CD16, CD19, CD36, CD56, CD123, TCRγ/δ and glycophorin A. Anti-biotin magnetic beads are then added for 15 minutes. Undesired cells are magnetically retained in a Magnetic-Activated Cell-Sorting (MACS®) column placed in a MACS® separator. The target cells pass through the column and are collected as the enriched, unlabeled, cell fraction, depleted of non-target cells. Flow cytometric analysis shows that more than 98% of the isolated cells are CD4+ cells.

#### Regulatory T cell isolation

Human regulatory T cells (Treg) isolation was performed using the CD4+CD25+ regulatory T cell Isolation kit (Miltenyi Biotec), according to manufacturer's instructions. Briefly, PBMCs and then CD4+ T cells are isolated as described above. Target cells, namely CD4+CD25+ cells, are then labelled with anti-CD25 beads. Cells are magnetically separated in a MACS® column placed in a MACS® separator. The flow-through fraction can be collected as negative fraction depleted of the labeled cells. The latter step is repeated once to enhance the yield and purity of the expected cell fraction. Flow cytometric analysis shows that more than 95% of the isolated cells are CD4+C25+ cells.

#### Neutrophils isolation

For all the experiments performed, human neutrophils were isolated from healthy donors' blood. Whole blood is first centrifuged to remove platelet-rich serum. The fraction containing the red blood cells, neutrophils and eosinophils were isolated by standard density gradient centrifugation using Ficoll-Paque PLUS (Amersham Biosciences, Uppsala, Sweden). From that point, immediately place the aliquots on ice. Then, the fraction is subjected to a lysis buffer (NH4CL 155 mM, KHCO3 10 mM, EDTA 0.1 mM) to remove red blood cells using a standard protocol. After 15 min on ice, cells were again centrifuged and the pellet was washed 3 times with PBS. Quality control is performed by cell counting after classical staining (Eosin; Methylene blue). And finally, the percentage of neutrophils was evaluated by light microscopy. Only preparations containing more than 95% of neutrophils were used. For subsequent analysis, isolated neutrophils cells were LPS-activated or not (1 µg/ml) (Sigma-Aldrich, St Louis, MO, USA) and inoculated with increasing amounts of purified H-1 PV (MOI 1, 5 and 25) or mock-treated from 24 h to 72 h.

#### Cell culture conditions

RPMI 1640 medium (Invitrogen, Paisley, UK) supplemented with 10% human AB serum (BioWest, Nuaillé, France), 2 mM L-Glutamine, 1 mM sodium pyruvate, 10 mM non essential amino acids, 10 mM HEPES, 50 U/mL streptomycin, 50 µg/mL gentamycin and 50 µM β-mercaptoethanol was used as standard culture medium. Cells were incubated at 37°C under controlled atmosphere (5% CO_2_ and 95% humidity) in a Hera Cell 150 incubator (Thermo Electron, Cergy Pontoise, France). When appropriate, PBMCs were activated using PHA (5 µg/mL). For CD4+ T cells activation, anti-CD3 (1,5 µg/mL) (Clinisciences, Montrouge, France) antibody was plate-bound by a 2 hours incubation at 37°C before the cultivation and soluble anti-CD28 antibody (100 ng/mL) (Clinisciences) was added extemporaneously.

### Flow cytometry

Cell immunophenotype was analyzed using EPICS® XL-MCL cytometer (Beckman Coulter, Fullerton, CA, USA) powered by EXPO 32 software. Cells were harvested, washed with phosphate-buffer saline (PBS) (Invitrogen) and labelled with appropriate fluorochrome-conjugated monoclonal antibodies (mAbs). Isotypic control mAbs were systematically used for markers setting. Data were analyzed and represented using Windows Multiple Document Interface for Flow Cytometry Software (WinMDI) (Scripps Research Institute, La Jolla, CA, USA).

Monoclonal antibodies (mAbs) directed against the relevant cell surface antigens were used to characterize main cells populations (PBMCs), cell activation status (PBMCs and CD4+ T cells) or cell phenotype (Treg cells). Anti-CD3-phycoerythrin(PE)-cyanin(Cy)5-mAb (BD Pharmingen, San Diego, CA, USA) was used to discriminate T cells from non T cells. Within T cells, anti-CD8-PE-mAb (Immunotech, Marseille, France) and anti-CD4-fluorescein isothiocyanate (FITC) or -PE-mAb (Immunotech) were respectively used to discriminate CD8 from CD4 populations. Within non T cells, anti-CD56-PE-mAb (BD Pharmingen) and anti-CD19-PE-mAb (BD Pharmingen) were respectively used to discriminate NK cells from B lymphocytes. Anti-CD4-PC5-mAb (BD Pharmingen), anti-CD25-PE-mAb (Miltenyi Biotec) and anti-CD127-FITC-mAb (Clinisciences) were used to characterize Treg cells phenotype. Finally, anti-CD30-PE-mAb (Immunotech) and anti-CD69-FITC-mAb (BD Pharmingen) were respectively used as late and early activation markers.

### Virus production and quantification, and cell infections

Briefly, NB-324K cells were transfected according to manufacturer's instructions using ExGen 500 transfection reagent (Euromedex, Souffelweyersheim, France) with a plasmid containing wild-type H-1 PV genome (pSR19, kindly provided by Pr. Jean Rommelaere, Heidelberg, Germany) [37] and allowing infectious viral particles to be generated. Cells were harvested 6 days after transfection by scraping, pelleted and lysed in 50 mM Tris-HCl/0,5 mM EDTA (pH 8,7) by two “freezing and thawing” cycles. Cell debris were removed by centrifugation and supernatant was collected for virus quantification (see below). MOLT 4 cells (ATCC CRL-1582™) were then infected with NB-324K-generated H-1 PV (Multiplicity of Infection or MOI = 0,01; expressed as the number of plate-forming unit/cell) for 3 days in order to produce large amounts of virus. Newly generated virions were collected as mentioned above and purified by iodixanol gradient centrifugation as previously described [38]. H-1 PV stock was quantified using Tissue Culture Infectious Dose 50 (TCID50) method. Briefly, NB-324K cells were seeded in 96-well plates and infected with serial dilutions of virus at the rate of 10 wells per dilution. Cells were incubated at 37°C (95% humidity, 5% CO_2_) for 4 days and then stained with Giemsa solution (Sigma-Aldrich, St Louis, MO, USA). Virus titers were calculated according to Reed and Muench method (1938). Part of the stock was UV-treated to inactivate virions and be used as a control. Cells (PBMCs, CD4+ T cells or Treg cells) were infected at various multiplicities of infection (MOI 1, 5 and 25). After cells cultivation (10^6^ cells/mL, whatever the cell culture tool is) and, if necessary, activation, virus was added at the rate of 10% of culture medium volume to allow proper spread and favor contact between cells and virus. A part of cells was also treated with PBS-MK, iodixanol or irradiated H-1 PV as controls. The inocula were not removed from cells. Iodixanol condition is further mentioned as “Mock”.

### Cell proliferation assay

Cells were incubated with [methyl-^3^H]-thymidine for the last 18 hours of culture and harvested on glass fibre filter (Printed Filtermat A, Wallac, Turku, Finland) using Tomtec harvester (Wallac). The filter was then sealed in a sample bag after drying and addition of scintillation liquid (Beckman Coulter). Radioactive thymidine, incorporated into replicated cellular DNA by proliferative cells, was detected by scintillation counting using a 1450 Trilux β-counter (Wallac) and measured in count per minute (cpm). Results are expressed as means of triplicate wells.

### Cell viability and cytotoxicity assays

In the course of infection, both cell viability and lysis were assessed using respectively CellTiter-Glo Luminescent Cell Viability assay and CytoTox-Glo Cytotoxicity assay according to manufacturer's instructions (Promega, Madison, WI, USA). The former measures the amount of ATP present in living cells and the latter evaluates the leak of a cellular marker from lysed cells cytoplasm to culture medium. Data were achieved using Centro LB 960 plate luminometer (Berthold, Thoiry, France) powered by MikroWin 2000 Software. Results are expressed in relative light unit (RLU) as means of triplicate wells.

### Cytokine detection

Culture supernatants were tested for cytokine detection by Enzyme-Linked ImmunoSorbent Assay (ELISA) method as previously described [39]. For each cytokine, the culture supernatant from about 10^5^ cells was used. In this study, IL-2, IL-4, IFN-γ and IL-10 release was investigated using sandwich ELISA based on colorimetric quantification. Antibodies pairs and standards were purchased at BD-Pharmingen. A_492 nm_ was measured using a multichannel spectrophotometer (Multiskan EX powered by Ascent Software, Thermo Electron). Results were expressed as means of duplicate wells.

### Western blotting

In the course of infection, cells were scraped directly in culture medium and washed with PBS. Cell pellets were then lysed (10 minutes on ice) in PY buffer consisting of 20 mM Tris-HCl, 50 mM NaCl, 5 mM EDTA, 1% Triton X-100, 0,02% sodium azide and a cocktail of proteases inhibitors (Roche, Basel, Switzerland). Cell debris were removed (20000 g, 15 minutes, 4°C), supernatants were collected and protein concentrations were measured using Bio-Rad Protein Assay (Bio-Rad, Marnes la Coquette, France) according to manufacturer's instructions. Total cell extracts (20 µg) were then analyzed by Western blotting. First, proteins were separated by SDS-PAGE electrophoresis using gradient pre-casts gels (4–12% gradient, Bis-Tris) (Invitrogen) and then transferred onto PVDF membrane (Millipore, Molsheim, France). The latter was blocked for 1 hour at room temperature in blocking buffer containing 0,2% Aurora™ blocking reagent (MP Biomedicals, Illkirch Graffenstaden, France), 0,1% Tween20 (Sigma-Aldrich) and PBS (1×), and incubated overnight at 4°C with primary antibodies directed against : NS1 protein (SP8 rabbit serum, 1∶5000) (Faisst et al., 1995), cleaved caspase 3 (1∶1000) (D175, 5AE1, Cell Signaling, Danvers, MA, USA), PARP (1∶1000) (H-250, Santa Cruz Biotechnology, Santa Cruz, CA, USA), β-actin (1∶5000) (sc-47778, Santa Cruz Biotechnology) and Erk2 (1∶1000) (sc-154, Santa Cruz Biotechnology). Membranes were washed with blocking buffer, incubated for 1 hour at room temperature with peroxydase-conjugated secondary antibodies (anti-mouse and anti-rabbit, 1∶10000) (GE Healthcare, Wauwatosa, WI, USA) and washed again with blocking buffer. Specific protein signals were visualized using Western Lightning® Plus-ECL, Enhanced Chemiluminescence Substrate kit (PerkinElmer, Boston, MA, USA).

### Regulatory T cell functionality assay

Mixed Leukocyte Reaction (MLR) assays were performed to test Treg cell ability to either proliferate under activation conditions or inhibit autologous PBMCs proliferative response. Autologous PBMCs, Treg cells or both cell types were cultured in round-bottom 96-well plates for 48 h. When appropriate, anti-CD3 (1,5 µg/mL) antibody was plate-bound by a 2 hour-incubation at 37°C before the cultivation and soluble anti-CD28 antibody (100 ng/mL) (Clinisciences) was added extemporaneously and/or cells were inoculated with H-1 PV as mentioned above. Cell proliferation was then assessed as described above. Results are expressed as means of triplicate wells.

### SCID Mice model

#### Animals

Homozygous CB-17 *scid/scid* (SCID) mice derived from breeding stocks provided by Dr M. Liberman (Standford University, Standford, CA), with the permission of Dr M. Bosma (Fow Chase Cancer Center, Philadelphia, PA), were bred and maintained in microisolator cages and used between 4 and 8 wk of age. The mice were kept under pathogen-free conditions without prophylactic administration of antibiotic.

#### Tumor xenotransplantation

Briefly, anesthetized mice were prepared for xenotransplantation by shaving the hair from a 2-cm2 area. Mice were subcutaneously xenotransplanted with nasopharyngeal carcinoma (NPC) tumors induced by C15 cells (Human primary tumor cells of NPC). The protocol was approved by the local Ethical Committee of the “Institut Pasteur de Lille”.

#### Human PBMC reconstitution

We waited for the development of visible tumors (about 6 to 8 weeks) before mice were splenectomized and reconstituted or not with human PBMC on day 0 (intraperitoneal injection of 50.10^6^ PBMC isolated from blood of healthy donor).

#### H-1 PV challenge

On day D0, the mice also received or not H-1 PV which is directly injected within the tumor (approximately 10 injection points for a final estimated concentration of MOI 0.1). Throughout the protocol mice received only one injection of H-1 PV.

Regular measurements of the tumor were performed from the immune reconstitution and H-1 PV challenge (at D3, D7, D9, D11, D15, D20, D22 and D23). During this period, some mice had to be sacrificed for ethical reasons. Finally, all mice were sacrificed at D23.

Under this experimental protocol, we made four separate groups of mice: (i) A control group of C15 xenotransplanted SCID mice which received neither PBMCs nor H-1 PV (n = 5); (ii) A group of xenotransplanted SCID mice receiving only PBMC (n = 5); (iii) A group of xenotransplanted SCID mice receiving only H-1 PV (n = 5); (iv) A group of xenotransplanted SCID mice receiving both PBMC and H-1 PV (n = 5).

### Statistical analysis

The relevance of the results was validated using the statistical rank sum Mann-Whitney test with Sigma-stat software.
